# Advancing Pediatric Surgery with Indocyanine Green (ICG) Fluorescence Imaging: A Comprehensive Review

**DOI:** 10.3390/children12040515

**Published:** 2025-04-16

**Authors:** Marco Di Mitri, Annalisa Di Carmine, Benedetta Zen, Edoardo Collautti, Cristian Bisanti, Simone D’Antonio, Michele Libri, Tommaso Gargano, Mario Lima

**Affiliations:** Pediatric Surgery Department, IRCCS Sant’Orsola Hospital, Alma Mater Studiorum University of Bologna, 40138 Bologna, Italy; marco.dimitri@studio.unibo.it (M.D.M.); benedetta.zen@studio.unibo.it (B.Z.); edoardo.collautti@studio.unibo.it (E.C.); cristian.bisanti@studio.unibo.it (C.B.); simone.dantonio@aosp.bo.it (S.D.); michele.libri@aosp.bo.it (M.L.); tommaso.gargano2@unibo.it (T.G.); mario.lima@unibo.it (M.L.)

**Keywords:** indocyanine green fluorescence, pediatric surgery, minimally invasive surgery, laparoscopy, robotic surgery, ICG

## Abstract

Background: Indocyanine green (ICG) fluorescence imaging has revolutionized pediatric surgery by enhancing precision, safety, and outcomes across various specialties. In recent years, its use has spread through the framework of pediatric surgery, where its ability to illuminate anatomical structures and pathological conditions has improved surgical outcomes. Methods: We conducted a systematic literature review following the Preferred Reporting Items for Systematic Reviews and Meta-Analysis (PRISMA) guidelines. The search was performed using the term “Indocyanine green” in all fields, including papers about pediatric patients (aged 0–18 years) published between January 2014 and July 2024. Results: This review systematically explores ICG applications, dosing regimens, timing of administration, and integration into modern surgical technologies, including robotics and minimally invasive platforms. ICG resulted in an excellent safety profile and enables the real-time visualization of anatomical structures and pathological conditions, proving invaluable in pediatric cases characterized by smaller anatomical dimensions and congenital anomalies. Conclusions: This review highlights ICG fluorescence imaging as an indispensable tool in pediatric surgery, offering transformative potential for improving surgical outcomes and patient safety. Despite its advantages, it is necessary to standardize dosing and timing protocols to maximize its utility. The aim of this review is to explore the various applications of ICG in pediatric surgery, report the dosage and administration times across different surgical fields, and establish best practices to guide its future use.

## 1. Introduction

The use of imaging technologies to enhance surgical precision has revolutionized modern medicine. Among these techniques, fluorescence-guided surgery (FGS) has gained prominence, particularly for its real-time visualization capabilities during surgical procedures [1]. Indocyanine green (ICG), a near-infrared fluorescent dye, is one of the most widely used agents in FGS. Introduced in 1957, ICG has a proven safety profile and diverse applications, including liver function assessment and retinal angiography. Its adoption in pediatric surgery has significantly improved outcomes by enabling better visualization of anatomical structures and pathological conditions [2]

ICG is a water-soluble dye that absorbs light at wavelengths of 600–900 nm and emits fluorescence detectable by specialized imaging systems. After intravenous injection, ICG binds to plasma proteins and is rapidly excreted into bile, making it particularly useful in liver function assessment and biliary anatomy visualization [3]. These properties are especially beneficial in pediatric surgery, where smaller anatomical structures and congenital anomalies present challenges.

In pediatric liver surgery, ICG fluorescence facilitates the identification of liver segments, bile ducts, and vascular structures, which is crucial for complex procedures involving congenital anomalies or tumors. Traditional methods, such as intraoperative ultrasound, have limitations, particularly when disease distorts anatomical landmarks. ICG fluorescence imaging provides a real-time, three-dimensional map of the liver, improving surgical precision and outcomes [4]

In pediatric oncology, ICG enhances tumor visualization, aiding in complete resection while preserving healthy tissues. By binding to proteins and accumulating in tumor tissues, ICG highlights cancerous areas under near-infrared light. This improves the accuracy of resections, reduces blood loss, and contributes to better outcomes in cancers like hepatoblastoma [5].

ICG fluorescence has also been incorporated into minimally invasive and robotic-assisted surgeries. Equipped with near-infrared cameras, robotic systems allow surgeons to visualize critical structures in real-time, reducing the risk of complications. This is particularly important in pediatric surgeries, where delicate tissues and small anatomical structures demand heightened precision [6].

Despite its advantages, the use of ICG fluorescence in pediatric surgery faces challenges, including the need for specialized imaging equipment and the determination of optimal dosage and timing. Pediatric patients’ unique metabolic rates and body compositions complicate dosage standardization, while the timing of ICG administration varies depending on the surgical application [7].

This review explores the diverse applications of ICG fluorescence in pediatric surgery, focusing on its dosing regimens and timing of administration. By synthesizing current data, it aims to establish best practices and guide future applications of ICG in this field.

## 2. Methods

This review was conducted to systematically explore the use of ICG in pediatric surgery, with a particular focus on its applications across different organ systems, the administered doses, and the timing of administration. The review employed a structured methodology to identify and analyze relevant studies, with the aim of providing comprehensive insights into the clinical use of ICG fluorescence in various pediatric surgical contexts.

### Search Strategy

**Figure 1:**
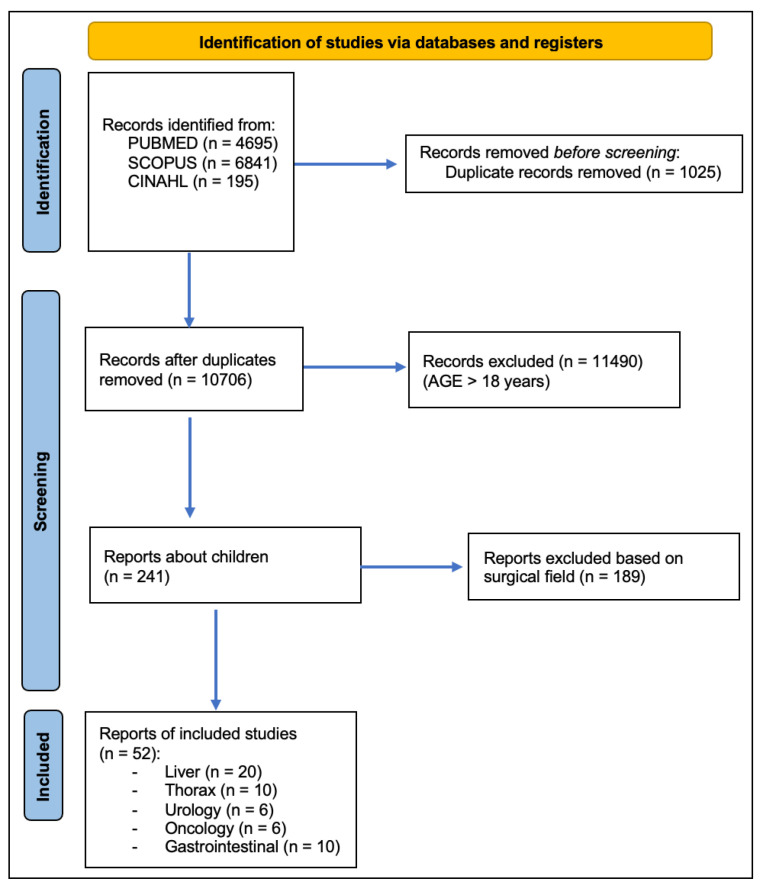
PRISMA statement.

We conducted a systematic literature review of PubMed, Scopus, and CINAHL in August 2024, following the Preferred Reporting Items for Systematic Reviews and Meta-Analysis (PRISMA) guidelines (Figure 1). We used the following MeSH terms during our search: “Indocyanine Green” and “Fluorescence” Boolean operators such as AND/OR were used to refine the search strategy; for example, “Indocyanine Green” OR “ICG”. We included papers published between January 2014 and July 2024. The search was limited to articles published in English and focused on studies involving human subjects, specifically pediatric patients (aged 0–18 years). All stages of the review process, including screening, selection, and data extraction, were conducted independently by two reviewers. Any discrepancies were resolved through discussion and consensus.

The inclusion criteria of the paper are as follows:

- Studies published between January 2014 and July 2024;
- Papers written in English;
- Studies involving pediatric patients (aged 0–18 years);
- Studies describing the use of indocyanine green (ICG) fluorescence imaging in pediatric surgical procedures.

The exclusion criteria are as follows:

- Non-clinical studies (animal models, in vitro research);
- Studies outside the field of pediatric surgery, including those focused exclusively on cardiac or neurosurgical procedures;
- Non-peer-reviewed publications (conference abstracts, editorials, letters);
- Studies lacking relevant data on ICG administration or surgical applications.

## 3. Results

### 3.1. Liver

#### 3.1.1. Cholecystectomy

**Figure 2:**
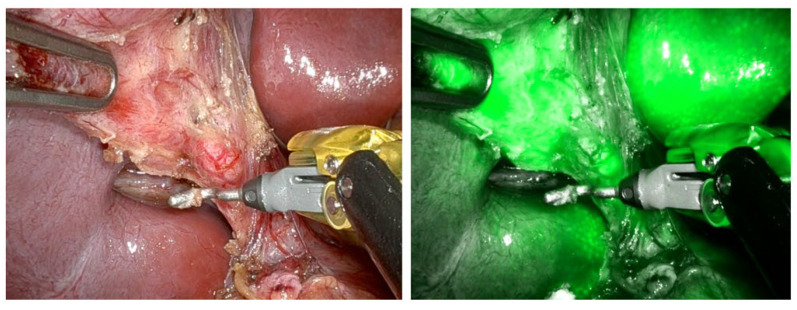
ICG use during cholecystectomy to allow the clear identification of de cystic duct.

The primary indication for ICG use in these studies was to improve the identification of the biliary system anatomy during laparoscopic cholecystectomy, particularly in cases where anatomical abnormalities or inflammation were present (Figure 2).

**Figure 3:**
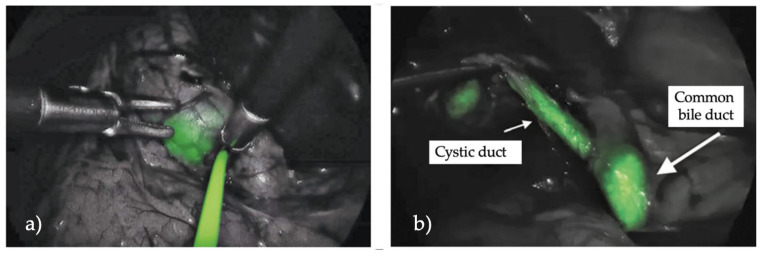
(**a**) Intraoperative cholangiography; (**b**) visualization of the cystic duct and the common bile duct.

In the study by Calabro et al., ICG fluorescence significantly improved the surgeon’s ability to identify the cystic duct (CD), common hepatic duct (CHD), and common bile duct (CBD), with visualization rates of 97.8%, 94%, and 96.1%, respectively; however, their study was an initial assessment, mainly focused on evaluating the safety and effectiveness of ICG, emphasizing the necessity for additional research [8]. Across further studies, ICG demonstrated a high success rate, with a 95.2% overall visualization rate reported by Esposito et al. [9] They also demonstrated in a retrospective study a statistically significant difference between the operative time with or without the ICG fluorescence (*p* = 0.001), which could improve intraoperative visualization during laparoscopic cholecystectomy [10,11]. Furthermore, ICG enables the identification of anatomical variants in 19% of patients, as seen in Daskalaki et al., who identified variations such as short cystic ducts and direct cystic duct insertions into the hepatic duct [12]. In terms of technical outcomes, there were no significant technical failures in the majority of the studies. Only one failure occurred due to poor video quality in a patient with high BMI and gangrenous cholecystitis, as reported by Daskalaki et al. Furthermore, ICG use resulted in a more precise anatomical visualization and improved surgical safety. The studies uniformly reported no adverse reactions or complications related to ICG administration, supporting its excellent safety profile. Additionally, the use of ICG in complex pediatric cases, as noted in Broderick et al. [13] in their univariable and multivariable logistic regression, contributed to reducing operative times and improved overall outcomes by aiding surgeons in the identification of key structures with statistically significant outcomes. In pediatric laparoscopic cholecystectomy, the use of indocyanine green (ICG) has been extensively studied to facilitate intraoperative visualization. ICG is primarily indicated for the visualization of biliary structures, especially during complex procedures or when anatomical variations are suspected (Figure 3).

The dosage of ICG varied between studies, with weight-based doses ranging from 0.35 mg/kg to 0.5 mg/kg and fixed doses of 2.5 mg and 7.5 mg. After converting weight-based doses using an average pediatric weight of 20 kg, the overall average ICG dose was 5.5 mg. The timing of administration is variable; the majority of studies recommend administration between 45 min and 18 h before surgery (Table 1).

#### 3.1.2. Hepatoblastoma

ICG fluorescence imaging has shown significant potential in the surgical treatment of hepatoblastoma, particularly by improving intraoperative visualization and permitting the identification of small or residual tumors. Across the studies, ICG was administered at doses ranging from 0.1 mg/kg to 0.5 mg/kg, with the timing of administration varying from 24 h to 90 h before surgery. Yamamichi et al. reported great outcomes in their case series in the visualization with ICG fluorescence for primary tumors, recurrent tumors, and multiple lung metastatic lesions [15]. ICG was primarily used to enhance precision in the detection of primary liver tumors and metastatic lung lesions in pediatric patients undergoing hepatoblastoma resection. The fluorescent imaging helped surgeons identify crucial anatomical features, with tumor sizes ranging from 1.2 mm to 15 mm, and to better delineate tumor boundaries. For example, Ryota Souzaki et al. reported the successful detection of residual tumors near the diaphragm and inferior vena cava, while Qiyang Shen et al. highlighted that 22 out of 23 patients exhibited clear tumor margins using ICG [16,17]. Also, Liu et al. showed that ICG was useful in locating tumors, determining surgical margins, and identifying small lesions that may not be visible on preoperative imaging [18] (Table 1).

#### 3.1.3. Biliary Atresia

ICG fluorescence imaging has also demonstrated its utility in pediatric patients undergoing surgery for biliary atresia, enhancing the precision of intraoperative decision-making. In a study by Yanagi et al., ICG (0.5 mg/kg) administered 24 h before surgery significantly improved the visualization of all bile ducts, as well as the liver surface, the transected plane of the porta hepatis, the hilar exudate, and the residual stump of the resected fibrous tissue, allowing for a more accurate dissection at the level of the hepatic hilum. This retrospective study reported that the normalization of postoperative bilirubin blood levels was more rapid in patients treated with ICG compared to those without its use (ratio 1.0 vs. 0.65), highlighting its impact on clinical outcomes [19].

Similarly, Hirayama et al. explored a different approach using a lower ICG dose of 0.1 mg/kg at the same time, demonstrating that real-time fluorescence improved bile duct visualization and facilitated better identification of the dissection level. This enabled surgeons to confirm the adequacy of bile flow, thereby optimizing resection margins and reducing postoperative complications [20].

Yi Zou Lim et al., administrating a dosage of 0.1 mg/kg, focused on the detection of ICG fluorescence in stool to assess biliary duct patency. They found a 97% accuracy in confirming patency in non-biliary atresia patients through stool fluorescence, whereas its absence indicated bile flow obstruction in biliary atresia cases [21].

Furthermore, Zhang et al., in their study (0.3 mg/kg 12 h before the procedure), showed that ICG was an effective method for diagnosing the causes of neonatal cholestasis, particularly biliary atresia, reducing surgical trauma and avoiding radiation exposure [22].

All these studies support the efficacy of ICG in providing clearer anatomical views during biliary atresia surgeries, leading to improved outcomes, particularly with regard to bile flow restoration and reduced postoperative complications (Table 1).

#### 3.1.4. Liver Transplant

In pediatric liver transplantation, ICG fluorescence imaging has proven to be a valuable tool for improving intraoperative guidance, particularly in complex procedures. In a case report by Li et al., a dose of 2.5 mg was administered intraoperatively to assist with the in-situ reduction of the liver’s segment III during a living donor liver transplantation using the Glissonean approach. The use of ICG fluorescence allowed for precise demarcation of hepatic segments, resulting in successful graft procurement without any graft-related complications [23].

In another case by Lemoine et al., ICG fluorescence (0.5 mg/kg) was used to detect a bile leak post-split liver transplantation. The real-time fluorescence imaging successfully localized a small bile leak, which was promptly repaired, leading to an excellent postoperative recovery for the patient. This highlights ICG’s effectiveness not only in graft procurement but also in managing post-transplant complications such as bile leaks [24].

A retrospective cohort study by Lu et al. using ICG at doses ranging from 0.004 mg/kg to 0.05 mg/kg during pure laparoscopic living donor left lateral sectionectomy reported no complications in either the donor or the recipient. ICG fluorescence facilitated the precise dissection and identification of critical anatomical structures, ensuring safe liver procurement and transplantation. Notably, the authors reported statistically significant improvements in operative time, warm ischemia time, and bile duct openings [25].

These studies underscore the utility of ICG in pediatric liver transplantation. Its applications include segmental liver demarcation for precise resection and the identification of complications like bile leaks, both of which significantly impact surgical outcomes. Furthermore, no adverse reactions or technical failures were reported in any of the studies, emphasizing the safety and reliability of ICG fluorescence in these settings (Table 1).

#### 3.1.5. Miscellanea

Indocyanine green (ICG) fluorescence has proven to be a valuable tool in various pediatric liver surgeries, particularly for anatomical navigation and the identification of critical structures. In the case report by Yamamoto et al., ICG (0.5 mg/kg) was administered four days prior to surgery in a child with undifferentiated embryonal sarcoma of the liver. The ICG fluorescence revealed a “rim-type” pattern, which helped to delineate the tumor margins without uptake within the tumor itself. This allowed for a precise resection, and no adverse events were reported. This case highlights the utility of ICG in rare liver tumors, providing clear boundaries for safe resection [26].

In a separate study by Masuya et al., ICG (0.6 mg/kg) was used intraoperatively to identify and preserve an aberrant right hepatic artery during laparoscopic surgery for congenital biliary dilatation. The real-time fluorescence provided by ICG was crucial in avoiding vascular injury in a complex anatomical scenario. The successful preservation of the artery and absence of adverse events further demonstrate the value of ICG in surgeries involving rare anatomical variants [27].

Both cases underscore the effectiveness and safety of ICG fluorescence in pediatric liver surgeries, particularly in guiding navigation during complex procedures or in patients with unusual anatomical features. The precise visualization provided by ICG can improve surgical outcomes and reduce the risk of complications (Table 1).

### 3.2. Thorax

#### 3.2.1. Chylothorax

Indocyanine green (ICG) fluorescence lymphography has shown significant potential in the treatment of pediatric patients with chylothorax, especially when conservative treatments fail. Chylothorax, a complication often seen after pediatric cardiac surgery, presents challenges due to the difficulty of identifying lymphatic leaks with traditional imaging techniques. However, ICG-based near-infrared fluorescence (NIRF) imaging offers a novel, minimally invasive solution to guide surgical interventions.

In the study by Tan et al., ICG lymphangiography successfully visualized abnormal lymphatic drainage patterns in a 5-week-old infant with postoperative chylothorax, allowing clinicians to guide the choice of pleurodesis for temporary symptom relief [28]. Similarly, Yokoyama et al. demonstrated that ICG could identify the leakage site during surgery, facilitating effective repair with fibrin sealant and polyglycolic acid (PGA) felt [29]. Both cases highlight ICG’s utility in real-time identification of lymphatic pathways and leak sites, particularly in complex cases where standard imaging methods may be insufficient.

ICG fluorescence has also been used to guide lymphatic mapping in patients with congenital lymphatic dysplasia, as demonstrated in Shibasaki et al.’s study. Their results indicated that ICG effectively visualized lymphatic channels and provided crucial guidance for successful surgical repair [30].

Shirotsuki et al. employed ICG-guided near-infrared (NIR) imaging for thoracoscopic navigation surgery to treat neonatal chylothorax, achieving direct visualization and ligation of chylous leakage sites. This technique allowed effective management of refractory chylothorax, with minimal complications and complete resolution in all cases [31]. Similar results were presented by Chang et al., who described the use of intraoperative ICG fluorescence lymphography to identify chylous leakage sites after congenital heart surgery. By injecting ICG subcutaneously in the inguinal region, the fluorescence highlighted a major leakage point, enabling precise ligation. The technique successfully resolved refractory chylothorax where previous surgical attempts had failed [32].

In all studies, the use of ICG proved to be safe, with no adverse reactions or significant complications reported. This highlights the potential of ICG fluorescence lymphography as a reliable tool for visualizing lymphatic structures and guiding the surgical treatment of chylothorax in pediatric patients. However, further studies are necessary to standardize ICG dosage and administration protocols to optimize outcomes across different clinical settings (Table 2).

#### 3.2.2. Pulmonary Surgery

**Figure 4:**
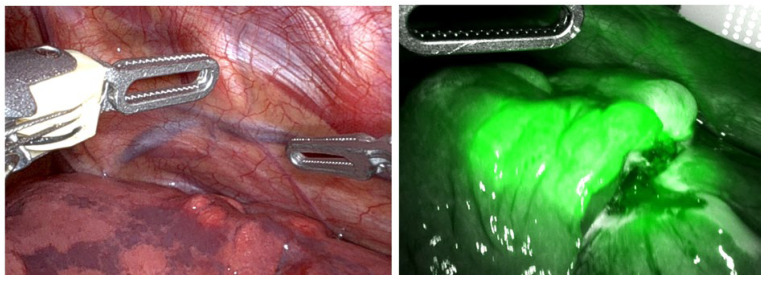
ICG use during a wedge pulmonary resection for inflammatory pseudotumor.

ICG fluorescence imaging has been explored in pediatric patients undergoing pulmonary metastasectomy, particularly for hepatoblastoma and sarcoma metastases. In a study by Yoshikawa et al., ICG (0.5 mg/kg) was administered 24 h before surgery and successfully localized a pulmonary nodule in a hepatoblastoma patient without any recurrence during a two-year follow-up [33]. Abdelhafeez et al. highlighted the successful localization of lung metastasis using ICG-guided fluorescence (1.5 mg/kg, the day before the surgery) in cases of hepatoblastoma and high-grade sarcoma, but this technology failed in cases such as neuroblastoma, adrenocortical carcinoma, myofibroblastic tumor, atypical cartilaginous tumor, and papillary thyroid carcinoma (Figure 4) [34].

Hamaji et al. demonstrated that doses ranging from 0.25 to 0.5 mg/kg could localize metastasis in only three patients affected by hepatocellular carcinoma, rhabdomyosarcoma, and breast adenocarcinoma out of 22 patients, highlighting its limitations in detecting pulmonary metastases, especially regarding solid tumors with higher incidence in adult patients [35]. In contrast, studies by Yamamichi et al. [15] and Kitagawa et al. [36] used ICG at a dose of 0.5 mg/kg, administrated, respectively, 3–4 days and 24 h before surgery, showing successful identification of metastases, particularly in hepatoblastoma patients, with no adverse events reported. Yoshida et al. reported a retrospective analysis of the application of ICG (0.5 mg/kg) used for patients with hepatoblastoma who underwent resection of pulmonary metastases, showing that histology-specific differences have an impact on tumor retention of ICG, emphasizing the necessity for further exploration. No significant safety concerns were reported, and ICG fluorescence proved to be a promising tool in cases of hepatic metastases [37]. Delgado et al. reported a case of successful visualization of bilateral lung metastases with ICG (0.5 mg/kg) in patients affected by hepatoblastoma who underwent thoracoscopic resection followed by living-donor liver transplantation [38].

Fung et al. presented a successful application of ICG fluorescence-guided pulmonary wedge resection in a 4-year-old child with a persistent pulmonary nodule following tuberculosis treatment. In this case, ICG fluorescence was useful for precisely defining the localization of a small, deep-seated nodule that may have been challenging to identify with conventional methods such as hookwire or methylene blue alone. The real-time imaging provided by ICG allowed for a prompt and accurate resection, minimizing tissue trauma and enabling the use of minimally invasive techniques [39]. (Table 2).

### 3.3. Urology

#### 3.3.1. Varicocelectomy

ICG fluorescence imaging has emerged as a valuable technique in pediatric varicocele treatment, specifically in lymphatic-sparing laparoscopic Palomo varicocelectomy. Zundel et al. reported promising results using a para-testicular injection of ICG (6.25 mg) to successfully visualize lymphatic vessels without harming the testes. Visualization of lymphatic vessels occurred promptly in most cases, and no adverse effects, such as testicular damage or scrotal staining, were observed [40].

Esposito et al. conducted a similar study with intratesticular ICG injection (2 mL of 5 mg/dL solution), and their results were similarly positive, with lymphatic vessels identified and spared in 100% of cases, no postoperative hydrocele, and no adverse reactions [41]. Both studies highlight ICG’s role in ensuring precise identification of lymphatic vessels, reducing the risk of postoperative complications such as hydrocele, which is often associated with techniques that do not spare lymphatic vessels.

The advantages of using ICG fluorescence in these procedures are supported by the fact that all studies reported 100% visualization success rates and no significant technical failures. The avoidance of postoperative hydrocele is especially notable, as traditional Palomo techniques can result in a 10–30% hydrocele rate due to the difficulty in identifying lymphatic vessels. Additionally, the safety profile of ICG is well-established, with no adverse effects or testicular pain reported in the patients across these studies [41].

In conclusion, ICG-guided fluorescence lymphography offers an effective and safe method for performing lymphatic-sparing varicocelectomy in pediatric patients. The consistent success across studies suggests that ICG should be considered a standard tool in this procedure to improve outcomes and reduce complications like hydrocele.

Furthermore, Tomita et al., in their paper, explored the use of ICG fluorescence angiography in laparo-endoscopic single-site varicocelectomy through intravenous administration. Using this technique, the authors showed the role of ICG in identifying spermatic arteries and veins, ensuring the accurate ligation of veins while preserving arteries and lymphatics, thereby reducing complications like hydrocele and recurrence [42] (Table 3).

#### 3.3.2. Urology Miscellanea

ICG fluorescence imaging has emerged as a valuable tool in pediatric surgery, providing enhanced anatomical visualization, improving surgical precision, and reducing postoperative complications. In laparoscopic partial nephrectomy for duplex kidneys, ICG was utilized by Esposito et al. at doses of 0.3 mg/kg/mL (intravenous) and 2.5 mg/mL (ureteral catheter) to delineate ureters, vasculature, ischemic boundaries, and residual kidney perfusion. This approach resulted in significantly reduced operative times and fewer complications compared to conventional techniques [44]. Similarly, in a case report about an onlay preputial island flap urethroplasty for hypospadias repair, the administration of ICG (0.15 mg/kg) enabled real-time assessment of tissue perfusion 80 s after injection, optimizing flap viability and reducing the risk of complications such as urethral stenosis and fistulas [45]. Furthermore, in robotic or laparoscopic renal cyst deroofing, ICG (0.35 mg/kg intraoperatively) facilitated the precise differentiation of vascularized renal tissue from non-vascularized cystic structures, enhancing procedural safety. The addition of fat-wadding techniques further reduced cyst recurrence rates [43]. Collectively, these studies underscore the versatility and clinical value of ICG fluorescence imaging in improving surgical outcomes in pediatric patients (Table 3).

### 3.4. Oncology

#### Nodes

ICG fluorescence imaging has emerged as a transformative innovation in oncological surgery, offering significant improvements in sentinel lymph node biopsy (SLNB), lymphatic mapping, and lymph node dissection across both pediatric and adult populations. Its ability to enhance visualization, improve detection rates, and minimize complications makes it a superior alternative to traditional methods such as blue dye and technetium-99 (99mTc). In melanoma, Fadel et al. reported that ICG achieved a detection rate of 96.7%, significantly outperforming blue dye, which achieved 89.6%. This enhanced visualization was particularly valuable in detecting sentinel lymph nodes (SLNs) in challenging anatomical regions, reducing the likelihood of undetected nodes and associated surgical complications [46]. Similarly, Knackstedt et al. demonstrated ICG’s utility in head and neck melanoma, a region with complex lymphatic drainage patterns, where ICG achieved a sensitivity of 91% and specificity of 100%, improving staging accuracy and guiding adjuvant therapy [47]. In pediatric oncology, ICG has proven especially impactful. Johnston et al. found that ICG was more effective than blue dye in SLNB, identifying more SLNs with a sensitivity of 84% and a positive predictive value of 91%. Moreover, its use reduced the need for prolonged anesthesia required for lymphoscintigraphy, making it a safer and more efficient option for pediatric patients [5]. Jeremiasse et al. further validated the use of ICG combined with technetium (ICG-TC) in SLNB for pediatric melanoma, sarcoma, and squamous cell carcinoma. Their study reported that 95% of SLNs were fluorescent, and ICG-TC identified additional nodes compared to radiotracers alone, demonstrating its superior accuracy and safety [48]. Beyond SLNB, Hirche et al. showcased ICG fluorescence imaging’s adaptability in lymphatic mapping across various solid tumors, including melanoma, sarcoma, gastric, colon, and breast cancers. They achieved a detection rate of 96% and sensitivity of 95.6%, underscoring its versatility and precision in surgical oncology [49]. Pio et al. explored the application of ICG in retroperitoneal lymph node dissection (RPLND) for pediatric patients with paratesticular rhabdomyosarcoma. Their novel single-port retroperitoneoscopic approach, combined with ICG guidance, enabled precise lymph node harvesting with no false negatives or complications. This technique also facilitated faster recovery, demonstrating the benefits of ICG in minimally invasive oncology procedures [50]. These studies collectively highlight the transformative potential of ICG fluorescence imaging in oncology. By providing real-time, enhanced visualization of lymphatic structures and sentinel nodes, ICG facilitates more precise surgical interventions, reduces the risk of complications, and improves patient outcomes. Its favorable safety profile, combined with its effectiveness across diverse anatomical and oncological scenarios, reinforces ICG’s critical role as a cornerstone in modern oncological surgery, particularly in pediatric and complex cases (Table 4).

### 3.5. Gastrointestinal Surgery

#### Miscellanea

ICG fluorescence imaging has demonstrated significant utility in enhancing precision and safety across a variety of pediatric gastrointestinal surgeries. Its applications include laparoscopic duodenal web excision, where intraduodenal ICG injection under near-infrared (NIR) light facilitates precise localization and successful removal of the web, avoiding complications [51]. In jejunal interposition for esophageal replacement, ICG/NIR imaging was used to assess graft perfusion at various stages, from selection to anastomosis, ensuring adequate blood flow and leading to adjustments in graft usage, including the abandonment of a poorly perfused graft, thus preventing postoperative ischemic complications [52]. In pediatric intestinal resections, a prospective trial revealed that ICG angiography had an impact on resection margins in 62% of cases, enhancing perfusion assessment and reducing anastomotic leaks and ischemia [53].

For esophageal surgery, ICG fluorescence was used to assess anastomotic perfusion and develop a risk stratification scorecard for poor outcomes like leaks or strictures, aiding surgical decision-making [54]. In complex colorectal surgeries such as cloacal reconstructions and Hirschsprung disease repairs, ICG-guided perfusion assessment led to surgical plan modifications in 31% of cases, including adjusting transection levels to ensure tissue viability, thereby reducing ischemic risks [55].

Li et al. also confirmed the role of ICG injected at the end of the rectum during laparoscopic-assisted anorectal pull-through surgery to detect, dissect, and cut the rectourethral fistulae, avoiding urological complications [56].

During laparoscopic Ladd’s procedures for intestinal malrotation, ICG imaging confirmed mesenteric perfusion, improving the safety of mesenteric root dissection and reducing the risk of volvulus recurrence [57]. In delayed anastomosis for long-gap esophageal atresia, ICG was employed to validate perfusion of the esophageal stumps, ensuring adequate blood flow and avoiding ischemic complications at the anastomotic site [58]. Lastly, in laparoscopic splenectomy and partial resection for splenic cysts, ICG facilitated vascular anatomy identification and highlighted thinning cyst walls, respectively, reducing intraoperative bleeding, improving dissection accuracy, and preserving splenic function. These findings were reported in a retrospective study by Esposito et al. [59].

A systematic review by Breukin et al. evaluated the safety and feasibility of indocyanine green fluorescence angiography (ICG-FA) in pediatric gastrointestinal surgery, considering various conditions such as esophageal atresia, intestinal volvulus, and intestinal malformation. Four studies demonstrated its utility in assessing intestinal perfusion and improving intraoperative decision-making, particularly in anastomotic blood flow and resection length. Eight studies on neonates showed that ICG-FA has a favorable safety profile with no serious adverse events. Limitations include small sample sizes and non-randomized designs, highlighting the need for larger prospective studies. Despite these limitations, ICG-FA shows promise as a safe and effective tool in pediatric surgery [60].

These studies collectively illustrate the transformative impact of ICG fluorescence imaging on pediatric surgery, enhancing real-time visualization, guiding surgical decisions, and improving postoperative outcomes while minimizing complications. This versatile tool has established itself as a cornerstone in advancing minimally invasive pediatric gastrointestinal procedures (Table 5).

## 4. Discussion

Indocyanine green (ICG) fluorescence imaging has emerged as a pivotal tool in pediatric surgery, enhancing intraoperative visualization and enabling real-time anatomical and functional assessment across various surgical specialties. Its applications span from gastrointestinal and hepatobiliary surgery to oncological and thoracic procedures, where precision and safety are paramount. ICG’s ability to provide detailed visualization of critical structures such as blood vessels, lymph nodes, and tumors has significantly improved surgical outcomes, particularly in pediatric cases where small anatomical features and congenital anomalies present unique challenges. In gastrointestinal surgery, ICG fluorescence has demonstrated significant utility in procedures such as laparoscopic duodenal web excision, jejunal interposition for esophageal replacement, and colorectal resections. It allows surgeons to assess perfusion accurately, adjust resection margins, and prevent complications like anastomotic leaks and ischemia. Similarly, in hepatobiliary procedures, such as cholecystectomy and liver transplantation, ICG improves the identification of bile ducts, hepatic segments, and vascular structures, enhancing the precision of resections and reducing operative risks. Furthermore, in oncology, ICG-guided fluorescence has transformed sentinel lymph node biopsy and tumor resection, providing clear tumor margins and better differentiation between healthy and malignant tissues. Studies have consistently reported its effectiveness in detecting small lesions and improving oncological outcomes, particularly in hepatoblastoma and metastatic pulmonary resections. ICG’s utility in thoracic surgery, particularly in the treatment of chylothorax and pulmonary nodules, highlights its versatility. ICG reduces the need for invasive interventions and facilitates minimally invasive approaches, offering precise lymphatic mapping and leak site identification. Additionally, its integration into robotic and minimally invasive platforms enhances its adaptability in complex procedures. Despite its widespread adoption, challenges remain. The reliance on specialized imaging equipment limits its accessibility in low-resource settings. Variability in dosing and timing of administration across studies underscores the need for standardization to optimize its efficacy. Moreover, while ICG is generally safe, rare adverse reactions necessitate careful patient monitoring, particularly in pediatric populations with varying metabolic profiles.

### 4.1. Complications and Adverse Effects of ICG Fluorescence

Indocyanine green (ICG) is a tricarbocyanine iodide dye with amphiphilic properties and it is prepared in an aqueous solution at pH 6.5 for administration via intravenous injection. Once introduced into the bloodstream, ICG rapidly associates with plasma proteins, predominantly remaining within the vascular compartment until it is taken up by the liver and subsequently excreted into the bile. Fluorescence imaging with indocyanine green (ICG) is a safe and effective technology [2]. In the studies analyzed, no patients reported the onset of adverse effects. In the literature, only a few case reports describe adverse reactions following the administration of ICG fluorescence. Chu et al. [61] reported a case of suspected intraoperative anaphylaxis following intravenous administration of ICG in an adult patient with a renal mass. The patient’s past medical history was significant for coronary artery disease with prior myocardial infarction, hypertension, and insulin-dependent diabetes mellitus. Sigley et al. [62] published a case report describing the extravasation of ICG after intravenous administration during an intestinal reanastomosis procedure. This resulted in a green discoloration along the volar aspect of the patient’s left forearm at the site of the intravenous catheter. The patient denied any discomfort at the site and exhibited no sensory, circulatory, or motor deficits. Management included the application of warm compresses over the area of infiltration and elevation of the left upper extremity. The discoloration gradually resolved and was no longer present by postoperative day 18.

### 4.2. Limitations

ICG fluorescence imaging faces challenges, including reliance on costly equipment, which limits its use in low-resource settings. Variability in dosing and timing protocols complicates standardization, affecting reproducibility, and the role of ICG decreases in deeper tissues due to limited fluorescence penetration.

### 4.3. Future Perspectives

The future of ICG fluorescence lies in advancing imaging systems for deeper tissue visualization and integrating artificial intelligence for enhanced accuracy. Standardized protocols for dosing and timing across procedures are essential. Innovations in dyes and cost-effective devices could expand access to underserved areas. ICG’s role in minimally invasive and robotic-assisted surgeries will grow, improving precision and outcomes. Research in pediatric and complex cases will further establish its transformative potential.

## 5. Conclusions

The evidence presented in this review focused attention on the transformative impact of ICG fluorescence imaging in pediatric surgery. Its ability to enhance intraoperative precision, improve safety, and optimize outcomes makes it an indispensable tool in modern surgical practice. While challenges in accessibility and standardization remain, the consistent success reported across diverse surgical specialties highlights its potential for broader integration into routine clinical practice. Future research should focus on establishing standardized protocols for dosing and timing, exploring its applications in emerging surgical technologies, and expanding its use to underserved regions. With ongoing advancements, ICG fluorescence imaging is expected to redefine the standards of surgical care, particularly in the field of pediatric surgery, where precision and safety are of utmost importance.

## Figures and Tables

**Table 1:** ICG used in liver surgery: dosing, time to administration, and visualized structures.

Liver				
Cholecystectomy				
Title	Authors	ICG Dosing	Time	Visualization
Indocyanine green (ICG) fluorescent cholangiography during laparoscopic cholecystectomy using RUBINA™ technology: preliminary experience in two pediatric surgery centers	Esposito et al. [9]	0.35 mg/kg	15.6 h	Biliary structures
Fluorescent cholangiography significantly improves patient outcomes for laparoscopic cholecystectomy	Broderick et al. [13]	7.5 mg	45 min	Cystic duct Hepatic ducts
Fluorescent cholangiography in laparoscopic cholecystectomy and the use in pediatric patients	Calabro et al. [8]	2.5 mg	Before surgical incision	Cystic duct Common hepatic duct Common bile duct
Twenty-five year experience with laparoscopic cholecystectomy in the pediatric population—from 10 mm clips to indocyanine green fluorescence technology: long-term results and technical considerations	Esposito et al. [10]	0.4 mg/kg	18 h	Biliary structures
Efficacy of indocyanine green (ICG) fluorescent cholangiography to improve intraoperative visualization during laparoscopic cholecystectomy in pediatric patients: a comparative study between ICG-guided fluorescence and standard technique	Esposito et al. [11]	0.35 mg/kg	15.6 h	Biliary structures
Near-infrared indocyanine green fluorescent cholangiography versus intraoperative cholangiography to improve safety in laparoscopic cholecystectomy for gallstone disease—a systematic review protocol	Pavel et al. [14].	0.5 mg/kg	2 h	Biliary structures
Indocyanine green (ICG) fluorescent cholangiography during robotic cholecystectomy: results of 184 consecutive cases in a single institution	Despoina Daskalaki [12]	2.5 mg	45 min	Biliary structures
**Hepatoblastoma**				
Clinical application of indocyanine green fluorescent imaging of hepatoblastoma	Yamamichi et al. [15]	0.5 mg/kg	3–4 days	Metastatic lesions
Navigation surgery using indocyanine green fluorescent imaging for hepatoblastoma patients	Souzaki et al. [16]	0.5 mg/kg	90.5 ± 33 h	Residual tumors near the diaphragm and vena cava
Effectiveness of indocyanine green fluorescence imaging in resection of hepatoblastoma	Shen et al. [17]	0.1 mg/kg	24–48 h	Tumor margins
Evaluating the clinical efficacy and limitations of indocyanine green fluorescence-guided surgery in childhood hepatoblastoma: a retrospective study	Liu et al. [18]	0.1 mg/kg	24h	Tumor margins
**Biliary Atresia**				
The outcome of real-time evaluation of biliary flow using near-infrared fluorescence cholangiography with indocyanine green in biliary atresia surgery	Yanagi et al. [19]	0.5 mg/kg	24 h	Hilar micro-bile ducts
Near-infrared fluorescence cholangiography with indocyanine green for biliary atresia. Real-time imaging during the Kasai procedure: a pilot study	Hirayama et al. [20]	0.1 mg/kg	24 h	Bile flow and the dissection level.
A pilot study for biliary atresia diagnosis: fluorescent imaging of indocyanine green in stool	Zou Lim et al. [21]	0.1 mg/kg	Variable timing	Biliary system patency
Role of indocyanine green-guided near-infrared fluorescence imaging in identification of the cause of neonatal cholestasis	Zhang et al. [22]	0.3 mg/kg	12 h	Distinguished biliary atresia from other causes of cholestasis
**Liver Transplant**				
Laparoscopic anatomic segment III procurement in pediatric living donor liver transplantation using real-time ICG fluorescence in situ reduction by the Glissonean approach	Li et al. [23]	2.5 mg/kg	During surgery	Graft vascularization
Indocyanine green fluorescence imaging as an adjunct for the localization of a bile leak after split liver transplantation	Lemoine et al. [24]	0.5 mg/kg	During surgery	Bile leak
The application of real-time indocyanine green fluorescence cholangiography in laparoscopic living donor left lateral sectionectomy	Lu et al. [25]	0.004–0.05 mg/kg	During surgery	Biliary anatomical structures and bile leak
**Miscellanea Liver Surgery**				
Rim-type indocyanine green fluorescence pattern in a child with undifferentiated embryonal sarcoma of the liver treated with navigation surgery	Yamamoto et al. [26]	0.5 mg/kg	4 days before surgery	Tumor margins
Using indocyanine green fluorescence in laparoscopic surgery to identify and preserve rare branching of the right hepatic artery in pediatric congenital biliary dilatation	Masuya et al. [27]	0.6 mg/kg	During surgery	Aberrant right hepatic artery

**Table 2:** ICG used in thoracic surgery: dosing, time to administration, and visualized structures.

Thorax				
Chylothorax				
Title	Authors	ICG Dosing	Time	Visualization
Investigational lymphatic imaging at the bedside in a pediatric postoperative chylothorax patient	Tan et al. [28]	0.05 mg	During surgery	Lymphatic leakage
Successful use of intraoperative ICG fluorescence lymphography and fibrin sealant with PGA felt for refractory chylous ascites in an infant: a novel procedure	Yokoyama et al. [29]	0.1 mL	During surgery	Lymphatic leakage
Evaluation of lymphatic dysplasia in patients with congenital pleural effusion and ascites using indocyanine green lymphography	Shibasaki et al. [30]	0.25 mg	During lymphography	Lymphatic leakage
Novel thoracoscopic navigation surgery for neonatal chylothorax using ICG fluorescent lymphography	Shirotsuki et al. [31]	0.025 mg	1 h	Lymphatic leakage
Intraoperative ICG fluorescence lymphography to detect chylous leakage sites after congenital heart surgery	Chang et al. [32]	0.5 mg	During surgery	Lymphatic leakage
**Pulmonary Surgery**				
Clinical application of indocyanine green in pediatric pulmonary metastases surgery	Yoshikawa et al. [33]	0.5 mg/kg	24 h	Metastasis localization
Feasibility of indocyanine green-guided localization of pulmonary nodules in children with solid tumors	Abdelhafeez et al. [34]	1.5 mg/kg	24 h	Metasasis localization
Near-infrared imaging using intravenous indocyanine green at a conventional dose to locate pulmonary metastases: a pilot study	Hamaji et al. [35]	0.25–0.5 mg/kg	12–24 h	Metastasis localization
Navigation using indocyanine green fluorescence imaging for hepatoblastoma pulmonary metastases surgery	Kitagawa et al. [36]	0.5 mg/kg	24 h	Metastasis localization
Clinicopathological study of surgery for pulmonary metastases of hepatoblastoma with indocyanine green fluorescent imaging	Yosida et al. [37]	0.5 mg/kg	24 h	Metastasis localization
Indocyanine green navigation in minimally invasive resection of multiple metachronous pulmonary metastases of hepatoblastoma	Carlos Delgado-Miguel [38]	0.5 mg/kg	24 h	Metastasis localization
ICG fluorescence-guided pulmonary wedge resection in a child	Fung et al. [39]	0.5 mL	1 h	Margins of a pulmonary nodule

**Table 3:** ICG used in urology: dosing, time to administration, and visualized structures.

Urology				
Varicocelectomy				
Title	Authors	ICG Dosing	Time	Visualization
Para-testicular injection of indocyanine green for laparoscopic immunofluorescence-guided lymphatic-sparing Palomo procedure	Zundel et al. [40]	6.25 mg	During surgery	Lymphatic vessels
Indocyanine green fluorescence lymphography: a new technique to perform lymphatic sparing laparoscopic Palomo varicocelectomy in children	Esposito et al. [41]	2 mL	During surgery	Lymphatic vessels
Indocyanine green angiography-assisted laparoendoscopic single-site varicocelectomy	Tomita et al. [42]	2.5 mg	During surgery	Artery and vein
**Miscellanea Urology Surgery**				
Laparoscopic or robotic deroofing guided by indocyanine green fluorescence and perirenal fat tissue injection in simple renal cysts	Esposito et al. [43]	0.35 mg/kg	During surgery	Margins of the cyst
Technical standardization of ICG-NIRF laparoscopic partial nephrectomy for duplex kidney	Esposito et al. [44]	0.3 mg/kg (intravenous) 2.5 mg (ureteral)	During surgery	Ureteral and vessel visualization
ICG-guided onlay preputial island flap urethroplasty for the single-stage repair of hypospadias in children: a case report	Paraboschi et al. [45]	0.15 mg/kg	During surgery	Flap vascularization

**Table 4:** ICG used in oncological surgery: dosing, time to administration, and visualized structures.

Oncologic Surgery				
Title	Authors	ICG Dosing	Time	Visualization
The use of indocyanine green and near-infrared fluorescence imaging in sentinel lymph node biopsy in cutaneous melanoma	Fadel et al. [46]	2.5 mg	During surgery	Visualization of the sentinel lymph node
Indocyanine green fluorescence imaging with lymphoscintigraphy improves the accuracy of sentinel lymph node biopsy in melanoma	Knackstedt et al. [47]	0.2–0.3 mL	During surgery	Visualization of the sentinel lymph node
Sentinel lymph node procedure in pediatric patients with melanoma, squamous cell carcinoma, or sarcoma using near-infrared fluorescence imaging with indocyanine green: a feasibility trial	Jeremiasse et al. [48]	0.25 mg	4–24 h	Visualization of the sentinel lymph node
Real-time lymphography by indocyanine green fluorescence: improved navigation for regional lymph node staging	Hirche et al. [49]	7 mg	During surgery	Visualization of the sentinel lymph node
Fluorescent-guided surgery and the use of indocyanine green sentinel lymph node mapping in the pediatric and young adult oncology population	Johnston et al. [5]	1.25 mg	During surgery	Visualization of the sentinel lymph node
Indocyanine green fluorescence-guided lymphadenectomy with single site retroperitoneoscopy in children	Pio et al. [50]	-	During surgery	Visualization of the sentinel lymph node

**Table 5:** ICG used in gastrointestinal surgery: dosing, time to administration, and visualized structures.

Gastrointestinal Surgery				
Title	Authors	ICG Dosing	Time	Visualization
Indocyanine green localization for laparoscopic duodenal web excision	Li et al. [51]	1.25 mg	During surgery	Localization of the web
Assessment of jejunal interposition perfusion using indocyanine green	Hall et al. [52]	0.2 mg/kg	During surgery	Perfusion evaluation
Indocyanine green fluorescence angiography in pediatric intestinal resections: a first prospective mixed methods clinical trial	Le-Nguyen et al. [53]	0.14 mg/kg	During surgery	Resection margins based on perfusion
Qualitative features of esophageal fluorescence angiography and anastomotic outcomes in children	Meisner et al. [54]	-	During surgery	Perfusion evaluation
Preliminary use of indocyanine green fluorescence angiography and value in predicting the vascular supply of tissues needed to perform cloacal, anorectal malformation, and Hirschsprung reconstructions	Rentea et al. [55]	0.1–0.3 mg/kg	During surgery	Perfusion evaluation
Indocyanine green fluorescence imaging localization: a helpful addition to laparoscopic dissection and division of rectourethral fistulae	Li et al. [56]	1.25 mg	During surgery	Localization of the rectourethral fistula
A safe and effective laparoscopic Ladd’s procedure technique involving the confirmation of mesenteric vascular perfusion by fluorescence imaging using indocyanine green: a case report of an infant	Sugita et al. [57]	-	During surgery	Perfusion evaluation
Feasibility of delayed anastomosis for long gap esophageal atresia in the neonatal period using internal traction and indocyanine green-guided near-infrared fluorescence	Onishi et al. [58]	0.5 mg/kg	During surgery	Perfusion evaluation
Twenty-five year experience with Minimally Invasive Splenectomy in Children: From Minilaparotomy to Use of Sealing Devices and Indocyanine Green Fluorescence Technology: Tips and tricks and Technical Considerations	Esposito et al. [59]	0.3 mg/kg/mL	During surgery	Vascular anatomy identification
Safety and feasibility of indocyanine green fluorescence Angiography in pediatric gastrointestinal surgery: a systematic review	Breuking et al. [60]	0.1–0.5 mg/kg	During surgery	Perfusion evaluation

## Data Availability

Not applicable.
